# Patient preferences for chiropractors’ attire: a cross-sectional study of UQTR university-based chiropractic clinic

**DOI:** 10.1186/s12998-025-00569-0

**Published:** 2025-01-31

**Authors:** Laurence Leduc, Jean Théroux, Caroline Marois, Geneviève Lavigne, Marc-André Blanchette

**Affiliations:** 1 Département de Chiropratique, Université du Québec À Trois-Rivières, 3351, Boulevard Des Forges, C.P. 500, Trois-Rivières, QC G9A 5H7 Canada; 2 Centre Hospitalier Universitaire Sainte-Justine, Montreal, QC Canada; 3 Université de Sherbrooke, Longueil, QC Canada; 4 Scientific Consultant, Vancouver, BC Canada

**Keywords:** Clothing, Patient satisfaction, Perception, Professional patient, Trust, Attire, Patient preference

## Abstract

**Background:**

A significant body of research has examined how the attire of physicians and nurses affects patients’ perceptions, preferences, and outcomes. However, limited research has focused on the clothing worn by other health professionals, such as chiropractors. The present study aims to explore patients’ preferences and perceptions of chiropractors’ attire.

**Methods:**

Using a cross-sectional image-based procedure, new patients to a university clinic were questioned regarding their preferences for four different attires (casual, formal, scrub, and white coat) worn by both a male and a female chiropractor. Patients also reported their perceptions in terms of chiropractors’ knowledge, trustworthiness, competence, professionalism, and comfortable for each photograph.

**Results:**

From August 10, 2022, to January 23, 2023, 75 new patients participated in the study. Results indicated a strong preference for scrubs for both male and female chiropractors. Chiropractors in scrubs were also seen as more knowledgeable, trustworthy, competent, and professional, and comfortable. This was closely followed by those wearing white coats and formal attire. Notably, the white coat worn by the female chiropractor received significantly more positive ratings than when worn by her male counterpart.

**Conclusion:**

In conclusion, our findings suggest that chiropractors’ attire influences patients’ perceptions and should be considered in the development of dress codes for public and private clinics. Further research is essential to understand better how the gender and age of care providers affect patient evaluations.

**Supplementary Information:**

The online version contains supplementary material available at 10.1186/s12998-025-00569-0.

## Introduction

Healthcare workers often wear uniforms for practical reasons or to be easily identifiable and recognizable.[1] Uniforms worn by healthcare providers can significantly influence various aspects of care. For example, healthcare uniforms contribute to the overall professional appearance of providers and can help instill confidence and trust.[2, 3] One widely recognized uniform in healthcare is the white coat, often worn by physicians. This piece of clothing is not without consequences, as it has been associated with elevated blood pressure in patients when in the presence of a healthcare provider.[4] This phenomenon is referred to as white coat syndrome or white coat hypertension. It is believed that the increase in blood pressure results from a stress response triggered in some patients by the presence of a healthcare professional, a medical environment, or the anticipation of a medical procedure.[5] Therefore, it is crucial to better understand the role of healthcare professionals’ attire and its influence, not only on blood pressure but also on the professional-patient relationship.

A large body of literature exists regarding the impacts of doctors’ and nurses’ dress codes on patients and their adherence to treatment.[6, 7] Although the white is often not mandatory,[8] patients overwhelmingly prefer seeing physicians dressed professionally in a white coat.[7, 9, 10] Physicians in white coats with formal attire are perceived as more experienced and professional,[11] and this attire leads to increased trust and patient satisfaction with care[7], as well as a greater likelihood of sharing sensitive information.[2] Similarly, nurses wearing scrubs are perceived as more skilled and knowledgeable,[12] while nurses in white pantsuit uniforms are viewed as more professional.[13].

Although less studied, uniforms and dress codes are also influential in other healthcare professions. For instance, physiotherapists’ attire impacts patients’ confidence and comfort, as well as the patient-therapist relationship.[14] Similar to physicians, physiotherapists wearing a white coat are generally perceived as more professional than those in scrubs or jeans.[15] However, Mercer and colleagues[15] noted that patients’ lifetime exposure (number of previous visits) to physiotherapists decreased the significance they placed on what the therapist was wearing. Age and gender also appear to be moderating factors to consider.[3, 6, 15].

Very little research exists regarding the optimal way of dressing for chiropractors. Results from an informal poll of 345 chiropractic physicians indicated that more than half typically wear smart casual attire (i.e., polo shirt and dress pants) when treating patients, while one in five reported wearing business attire.[16] This survey also showed that just over one in ten mentioned wearing a white coat. Chiropractors have indicated in a mixed-method study that their attire helps demonstrate their competence.[17] Limited research has been conducted from patients’ perspectives regarding chiropractors’ attire and its impact on patient satisfaction and adherence to treatment. Only one recent cross-sectional survey using an image-based online questionnaire could be found.[18] This study suggested that patients consider chiropractic students’ way of dressing important, indicating that wearing a white coat was not a necessity.[18].

Given the scarcity of studies regarding patients’ preferences and perceptions of chiropractors’ attire, the present study aims to address this question in a university chiropractic clinic. Specifically, the present study will use the same image-based procedure as employed by Théroux and colleagues [18] to explore patients’ perceptions of chiropractors’ knowledge, trustworthiness, competence, professionalism, and comfort based on their attire.

## Method

### Study design and procedure

The present study used a questionnaire-based cross-sectional design. Using consecutive sampling, all new adult patient of the *Université du Québec à Trois-Rivières* (UQTR) chiropractic clinic between August 10, 2022, and January 23, 2023, were invited by the clinic’s administrative assistants to complete a survey. Based on patients’ preferences, they either received an email invitation to the online survey or a paper copy of the questionnaire. The questionnaire began with a detailed description of the study to properly inform participants before they could consent. They were then invited to complete the questionnaire before seeing their chiropractor/intern wearing scrubs. Ethical approval from the UQTR Ethics Board for Research Involving Humans (CER-22–288-07.12) was obtained. To reach a sample representative of the 1400 new patients that seek care yearly at the university-based chiropractic clinic with a level of confidence of 80% and a margin of error of 5%, we estimated the required sample size at 147 participants.

### Questionnaire

The questionnaire used in this study was a French translation and adaptation of the one developed by Théroux and colleagues.[18] It was initially translated from English to Canadian French by a bilingual chiropractic intern, and the translation was subsequently revised by a bilingual epidemiologist experienced in cross-cultural adaptation of English questionnaires to Canadian French. Participants were presented with two sets of four photographs depicting a chiropractor in one of four types of attire: casual, formal, scrubs, or a white coat. One set featured a male chiropractor, while the other showcased a female chiropractor. To better reflect local options for clinical attire, the original Australian clothing choices (casual shorts, casual jeans, polo shirt, clinic shirt, and white coat) were replaced with Canadian equivalents: jeans and a white t-shirt (casual); black pants and a white dress shirt (formal); marine scrubs (scrubs); and black pants, a white dress shirt, and a white coat (white coat) (Table 3). Instead of asking how caring the photographed chiropractors made them feel, we instructed respondents to rate how competent they appeared. To reduce the length of the questionnaire, we focused solely on chiropractors’ attire rather than separately assessing preferences for students and supervising clinicians. Additionally, we replaced the 5-point rating scale with a 10-point scale to capture more subtle differences. The demographic questions were modified to align with Canadian guidelines.[19] The final translated and adapted questionnaire consisted of 49 questions divided into four sections (additional file S1).

In the first section, participants were invited to rate, on a scale ranging from 1 (very low) to 10 (excellent), how comfortable the photographed chiropractors made them feel and how knowledgeable, trustworthy, competent, and professional they appeared. These questions were asked subsequently for the four female and four male attires. To reduce potential biases, all eight photographs had the same background, and the chiropractors photographed displayed the same attitude and had similar physical appearance. In the second section of the questionnaire, participants were asked to rank the four attires, from favourite to least favourite, for the female and the male chiropractor separately. In the third section, participants were asked to indicate their level of agreement, on a Likert scale ranging from “strongly disagree” to “strongly agree”, with the following three statements: 1) the chiropractor’s attire is important to me, 2) the chiropractor’s attire influences my level of care satisfaction, and 3) the chiropractors should wear a white coat when seeing patients in the clinic. Finally, the fourth section of the questionnaire collected information on participants’ socio-demographic characteristics, including age, gender, educational status, and ethnicity.

### Data analysis

Descriptive statistics (means, standard deviations [SD], frequencies, and percentages) were computed for each question. A composite score was created by calculating the average of five domains (knowledgeability, trustworthiness, competence, professionalism, and making patients feel comfortable). Differences based on the gender of the chiropractor in participants’ ratings of the photographed attires on the five domains were assessed using paired-sample t-tests. Bivariate comparisons between participants’ preference for the chiropractors’ attire (most preferred) and participants’ characteristics were assessed using χ2 tests or ANOVAs when appropriate. A *p* value (2-sided) of ≤ 0.05 was deemed statistically significant. Analyses were conducted using IBM SPSS, version 27 (IBM SPSS Inc. Armonk. New York).

## Results

A total of 676 patients were invited to participate in the study, of which 75 completed the questionnaire (participation rate of 11.1%). Table 1 presents the socio-demographics of participants. The mean age was 38.5 years old; 37 were female, 34 were male, and 44% had a university education.

**Table 1:** Characteristics of the respondents

Characteristics	N (%)
Gender	
Female	37 (49.3)
Male	34 (45.3)
Other	1 (1.3)
Missing	3 (4.0)
Age; mean (SD)	38.5 (18.6)
Education	
Primary school	1 (1.3)
High school	13 (17.3)
College	24 (32.0)
University	33 (44.0)
Missing	4 (5.3)
Decline to answer	8 (2.5)
Ethnicity	
White, North American	59 (78.7)
White, Europe	2 (2.7)
Black, North American	1 (1.3)
Black, African	5 (6.7)
Decline to answer	3 (4.0)
Other	2 (2.7)
Missing	3 (4.0)

N = 75; SD: Standard deviation

### Participants’ perceptions of chiropractors’ skills and competence based on their attire

Table 2 presents the average scores of participants’ perceptions of chiropractors’ knowledge, trustworthiness, competence, professionalism, comfort, and total composite score for each attire photographed as well as the differences between chiropractors’ gender. The scrubs received the highest ratings of preference across all skills and competence domains compared to the other attires. The mean composite score for the scrubs was 8.4/10 for both the female and the male chiropractor. The attire with the lowest rating of preference for both the female and the male chiropractor was the casual attire with a mean composite score of 5.6/10. One significant difference was found. Specifically, on all domains and composite scores, the white coat was rated higher for the female chiropractor than for the male chiropractor.

**Table 2:** Rating of attire by domain and gender; mean (SD)

Domains	Casual			Formal			White coat			Scrub		
	Female	Male	*P* value	Female	Male	*P* value	Female	Male	*P* value	Female	Male	*P* value
Knowledgeable	5.7 (2.7)	5.5 (2.6)	0.191	6.9 (2.0)	7.1 (1.9)	0.151	7.5 (1.7)	7.0 (2.1)	0.002	8.2 (1.7)	8.3 (1.6)	0.535
Trustworthy	5.5 (2.6)	5.4 (2.5)	0.381	7.1 (1.9)	7.1 (2.0)	0.938	7.6 (1.7)	7.0 (2.0)	< 0.001	8.2 (1.6)	8.4 (1.6)	0.321
Competent	5.8 (2.6)	5.7 (2.6)	0.386	7.2 (1.8)	7.3 (1.9)	0.638	7.5 (1.7)	7.0 (2.1)	0.002	8.6 (1.5)	8.3 (1.7)	0.738
Professional	5.1 (2.8)	5.3 (2.6)	0.148	7.4 (1.9)	7.4 (1.9)	0.992	7.7 (1.8)	7.0 (2.1)	< 0.001	8.6 (1.5)	8.5 (1.6)	0.535
Comfortable	5.8 (2.8)	5.7 (2.5)	0.883	7.4 (1.9)	7.2 (2.0)	0.268	7.6 (1.9)	6.9 (2.2)	< 0.001	8.4 (1.6)	8.6 (1.6)	0.200
Mean score	5.6 (2.5)	5.5 (2.5)	0.769	7.2 (1.8)	7.2 (1.8)	0.800	7.6 (1.7)	7.0 (2.0)	< 0.001	8.4 (1.5)	8.4 (1.6)	0.556

### Participants’ preference regarding chiropractors’ attire

When specifically asked which attire they preferred, the majority of participants selected the scrubs (72% for the male chiropractor and 60% for the female chiropractor). Notably, for female chiropractor, nearly one in five participants (18.7%) preferred the white coat. The casual attire was the least preferred attire (57.3% for the male chiropractor and 69.3% for the female chiropractor) followed by the white coat (28% for the male chiropractor and 18.7% for the female chiropractor) (Table 3). Among the respondents’ characteristics, only their age was significantly associated with the preferred attire (Table 4). Respondents preferring a white coat were significantly older than those preferring the other attires.

**Table 3:** Preference for chiropractors’ attire; n (%)

	Casual	Formal	Scrub	White coat	Missing
Female					
Most preferred	1 (1.3)	12 (16.0)	45 (60.0)	14 (18.7)	*3 (4.0)*
Least preferred	52 (69.3)	3 (4.0)	1 (1.3)	14 (18.7)	*5 (6.7)*
Male					
Most preferred	0 (0.0)	9 (12.0)	54 (72.0)	7 (9.3)	*5 (6.7)*
Least preferred	43 (57.3)	3 (4.0)	1 (1.3)	21 (28.0)	*7 (9.3)*

**Table 4:** Bivariable association between the participants characteristics and their preference for chiropractors’ attire

Characteristics of respondents	Preference for chiropractors’ attire									
	Female					Male				
	Casual	Formal	White coat	Scrub	*P* value	Casual	Formal	White coat	Scrub	*P* value
Gender; n										
Female	0	5	9	22	0.789^a^	0	4	3	28	0.920^a^
Male	1	7	5	19		0	5	4	23	
Other	0	0	0	1		0	0	0	1	
Age; mean (SD)	–	35 (16)	52 (18)	33 (16)	0.007^b^	–	39 (20)	56 (14)	34 (16)	0.007^b^
Education; n										
Primary school	0	0	1	0	0.431^a^	0	0	1	0	0.051^a^
High school	0	1	4	8		0	2	2	9	
College	0	4	2	16		0	2	2	17	
University	1	7	6	18		0	4	1	27	

^a^χ2 test of trend

^b^ANOVA

### Participants’ opinions regarding chiropractors’ attire

A majority (50.6%) of participants considered that chiropractic attire is important, but only 21.3% reported that the chiropractor’s attire influences their level of satisfaction with the care they receive (Table 5). Additionally, up to one in five participants (21.4%) reported that chiropractors should wear a white coat when they see patients at the clinic (Table 5).

**Table 5:** Participants’ opinions regarding chiropractors’ attire; n (%)

	Strongly disagree	Disagree	Neither agree nor disagree	Agree	Strongly agree
The chiropractor attire is important to me *missing (n* = *2, 2.7%)*	7 (9.3%)	9 (12.0%)	19 (25.3%)	**28 (37.3%)**	10 (13.3%)
The chiropractor attire influences the level of satisfaction that I have for my care *missing (n* = *1, 1.3%)*	18 (24.0%)	16 (21.3%)	**24 (32.0%)**	15 (20.0%)	1 (1.3%)
Chiropractors should wear a white coat when they see patients at the clinic *missing (n* = *1, 1.3%)*	18 (24.0%)	**25 (33.3%)**	15 (20.0%)	11 (14.7%)	5 (6.7%)

Bold = mode

## Discussion

The aims of the present study were to explore new patients’ preferences at a university chiropractic clinic regarding the attire worn by chiropractic students, how the different attires influence patients’ perceptions of competence and skills, and whether student practitioners gender was associated with different patient perceptions. The present study used an image-based procedure. Overall, the findings suggest that the scrub was the preferred attire for both the female and the male chiropractor, while the casual attire was the least preferred. Furthermore, both the female and the male chiropractors were perceived as more knowledgeable, trustworthy, competent, professional, and able to make patients feel comfortable when wearing a scrub, followed by formal attire and a white coat. These results are in line with previous research using similar image-based procedure with physiotherapists [15] and student chiropractors [18].

To the best of our knowledge, this study is only the second to investigate the impact of chiropractors’ attire on patients. Théroux and colleagues[18] reported that student chiropractors wearing the clinic’s shirt, or a white coat, were highly rated. One important limitation of Théroux et al.’s study,[18] especially in light of the present results, is their omission of the scrub as an attire for chiropractors. Indeed, in the present study, the scrub was the overwhelmingly preferred attire. However, chiropractors wearing the white coat and the formal attires were also judged as knowledgeable, trustworthy, competent, professional, and able to make patients feel comfortable. Many previous studies with physicians and nurses have also found the scrub and the white coat to be very acceptable to patients.[7, 9, 10, 12, 13].

Interestingly, in the present study, the female chiropractor wearing a white coat was perceived as more knowledgeable, trustworthy, competent, professional, and able to make the patient feel comfortable than the male chiropractor wearing the same attire. Similarly, the white coat was the most preferred attire for twice as many participants when worn by the female chiropractor (18.7%) than when worn by the male chiropractor (9.3%). This departs from Mercer and colleagues (2008) who found that more educated participants found the white coat on a female physiotherapist less appropriate than on a male physiotherapist. When comparing patients’ perceptions of physicians’ attire in the emergency department, Li and Haber [20] reported no differences based on the gender of the provider. However, Kurihana and colleagues [21] reported that patients perceived female physicians dressed in formal attire without a white coat as more inappropriate than a similarly dressed male physician. Future research appears necessary to replicate the present findings and to determine if patients systematically have a greater preference for the white coat when worn by female chiropractors than by male chiropractors.

While most participants in the present study reported that a chiropractor’s attire is important, only 21% reported that their level of satisfaction with the care they receive is influenced by the chiropractor’s clothing. This finding aligns with the results of Hossler and colleagues,[22] who reported no significant changes in participants’ level of satisfaction with care when an outpatient dermatology clinic changed its policy from formal attire to fitted scrubs. As suggested by Hossler and colleagues,[22] factors other than the attire worn by the care provider likely influence patients’ satisfaction with the care they receive, such as their communication style, empathy, and demonstrated skills.

Emerging evidence suggests that contextual factors—including patients’ beliefs and characteristics, practitioners’ beliefs and characteristics, the patient-practitioner relationship, the therapeutic setting or environment, and treatment characteristics—may enhance conservative care for chronic low back pain.[23–25] The influence of these factors might not always be interpreted as a conscious cognitive process by patients. Notable contextual factors identified by Sherriff and colleagues,[23] include addressing maladaptive illness beliefs, providing verbal suggestions to influence expectations for symptom change, utilizing visual or physical cues to indicate pain-relieving treatment properties, and fostering positive communication, such as empathy, to strengthen the therapeutic alliance. While little is known about the mechanisms of action regarding these contextual factors, Sherriff and colleagues suggest that modifying more than one factor may yield greater effectiveness. Our results imply that a chiropractor’s attire may serve as a contextual factor influencing trust. Trust is consistently identified as a factor that positively affects the therapeutic alliance, potentially leading to improved pain outcomes and increased treatment satisfaction for individuals undergoing physical rehabilitation for chronic musculoskeletal pain.[24, 25] Future research should investigate the interplay between clinical attire, trust, therapeutic alliance, and other contextual factors to enhance our understanding of their influence of clinical outcomes.

### Strengths and limitations

The present study has several strengths and limitations that deserve mention. First, the lower-than-expected response rate might have influenced the representativity of our sample. The obtained sample size of 75 respondents allows us to make inferences about the target population with a confidence level of 80% and a margin of error of 7.2%. Moreover, the generalizability outside of the province of Quebec might be limited given attire is very much a cultural signal.[26] Second, using an image-based procedure offered a standardized target to be evaluated by participants. However, only one male and one female chiropractor were photographed, which may have introduced other biases. One potential factor that may have influenced participants’ evaluations is the young age of the chiropractors photographed. The study took place in a university clinic where student chiropractors treat patients but where more senior chiropractors are also involved with patients. Given that patients’ age does influence their perceptions of the appropriateness of a professional’s attire [3, 15] it is possible that the age of the provider also influences patients’ perceptions. Another limitation of the present study is that all the chiropractors of the university clinic where participants were recruited had to wear scrubs due to the COVID-19 pandemic. Chiropractors in private settings have more liberty to wear the attire of their choice. Thus, the environment where participants were recruited, and the presence of chiropractors wearing scrubs in that particular clinic may have skewed the preference for this specific attire. Prior knowledge of the intern by the patients may also influence respondents’ perceptions. We did not collect information on whether the new patients were familiar with the interns before their first appointment. However, interns are required to see at least 40 new patients during their internship, with a maximum of 5 patients allowed to come from ‘family and friends,’ which may limit this potential source of bias. Finally, the paper version of the survey presented black and white pictures, while the web-based questionnaire presented colour pictures. Also, pictures were not taken by a professional photographer. The quality of the pictures may have affected patients’ perceptions.

## Conclusion

In sum, the aims of the present study were to explore patients’ perceptions and preferences regarding the attire worn by chiropractors in a university clinic. Overall, results suggest that scrubs are the most preferred attire, while casual attire is the least preferred. Chiropractors wearing scrubs received the highest scores of perceived knowledgeability, trustworthiness, competence, professionalism, and ability to make patients feel comfortable. The formal attire and the white coat also received high scores on these perceived competence and skill domains. More research is nonetheless needed to better understand how a number of moderators, such as providers’ and patients’ age, lifetime exposure to chiropractic treatments, and clinic settings, influence patients’ perceptions and preferences.

## Supplementary Information

Additional file 1.

## Data Availability

The data that support the findings of this study are not openly available but are available from the corresponding author upon reasonable request within 6 months of the manuscript publication. After that time period the data will be destroyed as specified within our ethical certification application.
