# Supplementary material for: Patient preferences for chiropractors’ attire: a cross-sectional study of UQTR university-based chiropractic clinic
Source: Chiropr Man Therap. 2025 Jan 31;33:5. doi: 10.1186/s12998-025-00569-0 (PMC11786509; doi:10.1186/s12998-025-00569-0)
Supplement: Supplementary file 1 — Additional file 1. [file 12998_2025_569_MOESM1_ESM.pdf]

Attitude des patients envers l'uniforme clinique

Nom : ______________________________

Code permanent : ______________

Date d'examen : ____________

Session : ______________

Sigle : ________

Groupe : ___

SECTION 1 / 4 : SECTION A – ÉVALUATION DE LA TENUE VESTIMENTAIRE DES CHIROPRATICIENS

Évaluer la tenue vestimentaire des chiropraticiens pour chaque question. Sur une échelle de 1 à 10, choisissez le chiffre qui correspond le mieux à votre réponse.

1.1.

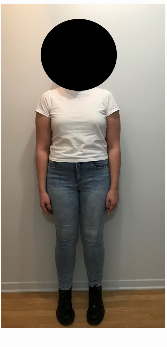

1. Comment évaluez-vous le niveau de connaissance de cette chiropraticienne?
   - 1 (très faible)
   - 2
   - 3
   - 4
   - 5
   - 6
   - 7
   - 8
   - 9
   - 10 (excellent)
2. Comment évaluez-vous le niveau de confiance que vous avez envers cette chiropraticienne?
   - 1 (très faible)
   - 2
   - 3
   - 4
   - 5
   - 6
   - 7
   - 8
   - 9
   - 10 (excellent)
3. Comment évaluez-vous le niveau de compétence de cette chiropraticienne?
   - 1 (très faible)
   - 2
   - 3
   - 4
   - 5
   - 6
   - 7
   - 8
   - 9
   - 10 (excellent)
4. Comment évaluez-vous le professionnalisme de cette chiropraticienne?
   - 1 (très faible)
   - 2
   - 3
   - 4
   - 5
   - 6
   - 7
   - 8
   - 9
   - 10 (excellent)
5. Dans quelle mesure cette chiropraticienne vous met-elle à l'aise?
   - 1 (très faible)
   - 2
   - 3
   - 4
   - 5
   - 6
   - 7
   - 8
   - 9
   - 10 (excellent)

1.2.

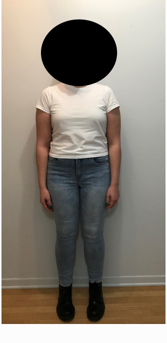

1. Comment évaluez-vous le niveau de connaissance de cette chiropraticienne?
   - 1 (très faible)
   - 2
   - 3
   - 4
   - 5
   - 6
   - 7
   - 8
   - 9
   - 10 (excellent)
2. Comment évaluez-vous le niveau de confiance que vous avez envers cette chiropraticienne?
   - 1 (très faible)
   - 2
   - 3
   - 4
   - 5
   - 6
   - 7
   - 8
   - 9
   - 10 (excellent)
3. Comment évaluez-vous le niveau de compétence de cette chiropraticienne?
   - 1 (très faible)
   - 2
   - 3
   - 4
   - 5
   - 6
   - 7
   - 8
   - 9
   - 10 (excellent)
4. Comment évaluez-vous le professionnalisme de cette chiropraticienne?
   - 1 (très faible)
   - 2
   - 3
   - 4
   - 5
   - 6
   - 7
   - 8
   - 9
   - 10 (excellent)
5. Dans quelle mesure cette chiropraticienne vous met-elle à l'aise?
   - 1 (très faible)
   - 2
   - 3
   - 4
   - 5
   - 6
   - 7
   - 8
   - 9
   - 10 (excellent)

1.3.

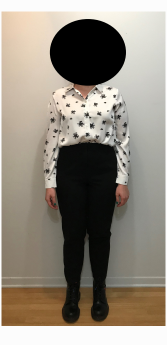

1. Comment évaluez-vous le niveau de connaissance de cette chiropraticienne?
   - 1 (très faible)
   - 2
   - 3
   - 4
   - 5
   - 6
   - 7
   - 8
   - 9
   - 10 (excellent)
2. Comment évaluez-vous le niveau de confiance que vous avez envers cette chiropraticienne?
   - 1 (très faible)
   - 2
   - 3
   - 4
   - 5
   - 6
   - 7
   - 8
   - 9
   - 10 (excellent)
3. Comment évaluez-vous le niveau de compétence de cette chiropraticienne?
   - 1 (très faible)
   - 2
   - 3
   - 4
   - 5
   - 6
   - 7
   - 8
   - 9
   - 10 (excellent)
4. Comment évaluez-vous le professionnalisme de cette chiropraticienne?
   - 1 (très faible)
   - 2
   - 3
   - 4
   - 5
   - 6
   - 7
   - 8
   - 9
   - 10 (excellent)
5. Dans quelle mesure cette chiropraticienne vous met-elle à l'aise?
   - 1 (très faible)
   - 2
   - 3
   - 4
   - 5
   - 6
   - 7
   - 8
   - 9
   - 10 (excellent)

1.4.

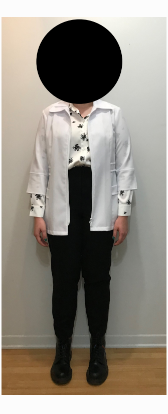

1. Comment évaluez-vous le niveau de connaissance de cette chiropraticienne?
   - 1 (très faible)
   - 2
   - 3
   - 4
   - 5
   - 6
   - 7
   - 8
   - 9
   - 10 (excellent)
2. Comment évaluez-vous le niveau de confiance que vous avez envers cette chiropraticienne?
   - 1 (très faible)
   - 2
   - 3
   - 4
   - 5
   - 6
   - 7
   - 8
   - 9
   - 10 (excellent)
3. Comment évaluez-vous le niveau de compétence de cette chiropraticienne?
   - 1 (très faible)
   - 2
   - 3
   - 4
   - 5
   - 6
   - 7
   - 8
   - 9
   - 10 (excellent)
4. Comment évaluez-vous le professionnalisme de cette chiropraticienne?
   - 1 (très faible)
   - 2
   - 3
   - 4
   - 5
   - 6
   - 7
   - 8
   - 9
   - 10 (excellent)
5. Dans quelle mesure cette chiropraticienne vous met-elle à l'aise?
   - 1 (très faible)
   - 2
   - 3
   - 4
   - 5
   - 6
   - 7
   - 8
   - 9
   - 10 (excellent)

1.5.

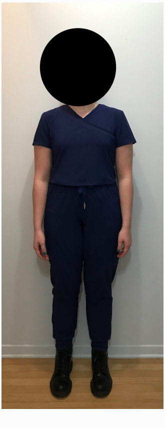

1. Comment évaluez-vous le niveau de connaissance de cette chiropraticienne?
   - 1 (très faible)
   - 2
   - 3
   - 4
   - 5
   - 6
   - 7
   - 8
   - 9
   - 10 (excellent)
2. Comment évaluez-vous le niveau de confiance que vous avez envers cette chiropraticienne?
   - 1 (très faible)
   - 2
   - 3
   - 4
   - 5
   - 6
   - 7
   - 8
   - 9
   - 10 (excellent)
3. Comment évaluez-vous le niveau de compétence de cette chiropraticienne?
   - 1 (très faible)
   - 2
   - 3
   - 4
   - 5
   - 6
   - 7
   - 8
   - 9
   - 10 (excellent)
4. Comment évaluez-vous le professionnalisme de cette chiropraticienne?
   - 1 (très faible)
   - 2
   - 3
   - 4
   - 5
   - 6
   - 7
   - 8
   - 9
   - 10 (excellent)
5. Dans quelle mesure cette chiropraticienne vous met-elle à l'aise?
   - 1 (très faible)
   - 2
   - 3
   - 4
   - 5
   - 6
   - 7
   - 8
   - 9
   - 10 (excellent)

1.6.

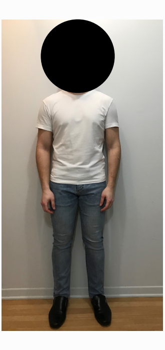

1. Comment évaluez-vous le niveau de connaissance de ce chiropraticien?
   - 1 (très faible)
   - 2
   - 3
   - 4
   - 5
   - 6
   - 7
   - 8
   - 9
   - 10 (excellent)
2. Comment évaluez-vous le niveau de confiance que vous avez envers ce chiropraticien?
   - 1 (très faible)
   - 2
   - 3
   - 4
   - 5
   - 6
   - 7
   - 8
   - 9
   - 10 (excellent)
3. Comment évaluez-vous le niveau de compétence de ce chiropraticien?
   - 1 (très faible)
   - 2
   - 3
   - 4
   - 5
   - 6
   - 7
   - 8
   - 9
   - 10 (excellent)
4. Comment évaluez-vous le professionnalisme de ce chiropraticien?
   - 1 (très faible)
   - 2
   - 3
   - 4
   - 5
   - 6
   - 7
   - 8
   - 9
   - 10 (excellent)
5. Dans quelle mesure cet chiropraticien vous met-il à l'aise?
   - 1 (très faible)
   - 2
   - 3
   - 4
   - 5
   - 6
   - 7
   - 8
   - 9
   - 10 (excellent)

1.7.

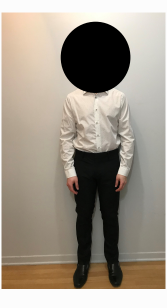

1. Comment évaluez-vous le niveau de connaissance de ce chiropraticien?
   - 1 (très faible)
   - 2
   - 3
   - 4
   - 5
   - 6
   - 7
   - 8
   - 9
   - 10 (excellent)
2. Comment évaluez-vous le niveau de confiance que vous avez envers ce chiropraticien?
   - 1 (très faible)
   - 2
   - 3
   - 4
   - 5
   - 6
   - 7
   - 8
   - 9
   - 10 (excellent)
3. Comment évaluez-vous le niveau de compétence de ce chiropraticien?
   - 1 (très faible)

- 2
- 3
- 4
- 5
- 6
- 7
- 8
- 9
- 10 (excellent)

4. Comment évaluez-vous le professionnalisme de ce chiropraticien?

- 1 (très faible)
- 2
- 3
- 4
- 5
- 6
- 7
- 8
- 9
- 10 (excellent)

5. Dans quelle mesure cet chiropraticien vous met-il à l'aise?

- 1 (très faible)
- 2
- 3
- 4
- 5
- 6
- 7
- 8
- 9
- 10 (excellent)

1.8.

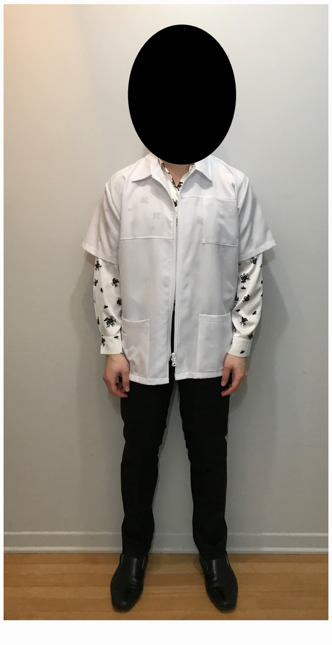

1. Comment évaluez-vous le niveau de connaissance de ce chiropraticien?

- 1 (très faible)
- 2
- 3
- 4
- 5
- 6
- 7
- 8
- 9
- 10 (excellent)

2. Comment évaluez-vous le niveau de confiance que vous avez envers ce chiropraticien?

- 1 (très faible)
- 2
- 3
- 4
- 5
- 6
- 7
- 8
- 9
- 10 (excellent)

3. Comment évaluez-vous le niveau de compétence de ce chiropraticien?

- 1 (très faible)
- 2
- 3
- 4
- 5
- 6
- 7
- 8
- 9
- 10 (excellent)

4. Comment évaluez-vous le professionnalisme de ce chiropraticien?

- 1 (très faible)
- 2
- 3
- 4
- 5
- 6
- 7
- 8
- 9
- 10 (excellent)

5. Dans quelle mesure cet chiropraticien vous met-il à l'aise?

- 1 (très faible)
- 2
- 3
- 4
- 5
- 6
- 7
- 8
- 9
- 10 (excellent)

1.9.

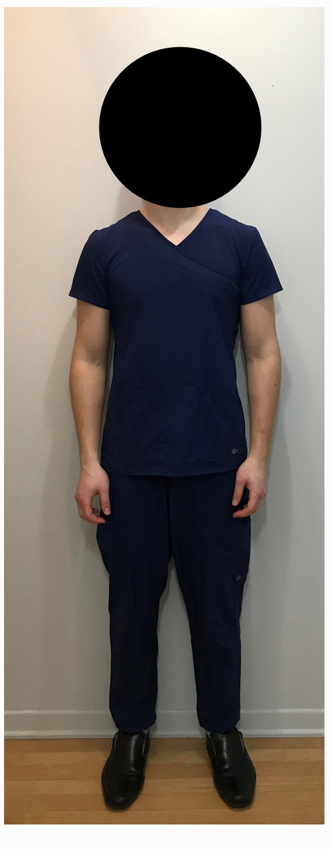

1. Comment évaluez-vous le niveau de connaissance de ce chiropraticien?

- 1 (très faible)
- 2
- 3
- 4
- 5
- 6
- 7
- 8
- 9
- 10 (excellent)

2. Comment évaluez-vous le niveau de confiance que vous avez envers ce chiropraticien?

- 1 (très faible)
- 2
- 3
- 4
- 5
- 6
- 7
- 8
- 9
- 10 (excellent)

3. Comment évaluez-vous le niveau de compétence de ce chiropraticien?

- 1 (très faible)
- 2
- 3
- 4
- 5
- 6
- 7
- 8
- 9
- 10 (excellent)

4. Comment évaluez-vous le professionnalisme de ce chiropraticien?

- 1 (très faible)
- 2
- 3
- 4
- 5
- 6
- 7
- 8
- 9
- 10 (excellent)

5. Dans quelle mesure cet chiropraticien vous met-il à l'aise?

- 1 (très faible)
- 2
- 3
- 4
- 5
- 6
- 7
- 8
- 9
- 10 (excellent)

SECTION 2 / 4 : SECTION B – PRÉFÉRENCE D'UNE TENUE VESTIMENTAIRE

2.1. Quelle chiropraticienne préférez-vous?

Classer les images des chiropraticiennes en ordre en fonction de votre perception de leur niveau de connaissances et compétences.

(1 = tenue préférée et 4 = tenue la moins appréciée)

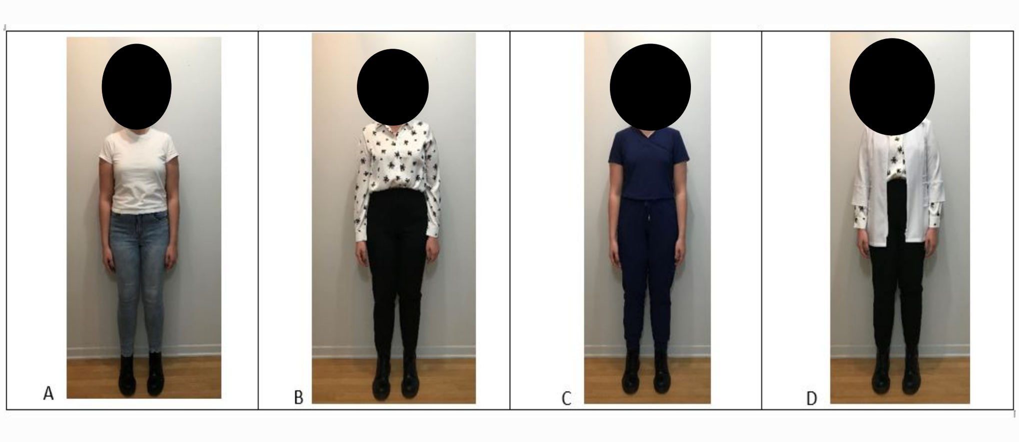

Classement

1.

- A
- B
- C
- D

Tenue préférée

2.

- A
- B
- C
- D

3.

- A
- B
- C
- D

4.

- A
- B
- C
- D

Tenue la moins préférée

2.2. Quel chiropraticien préférez-vous?

Classer les images des chiropraticiens suivants en fonction de votre perception de leur niveau de connaissances et compétences.

(1 = tenue préférée et 4 = tenue la moins appréciée)

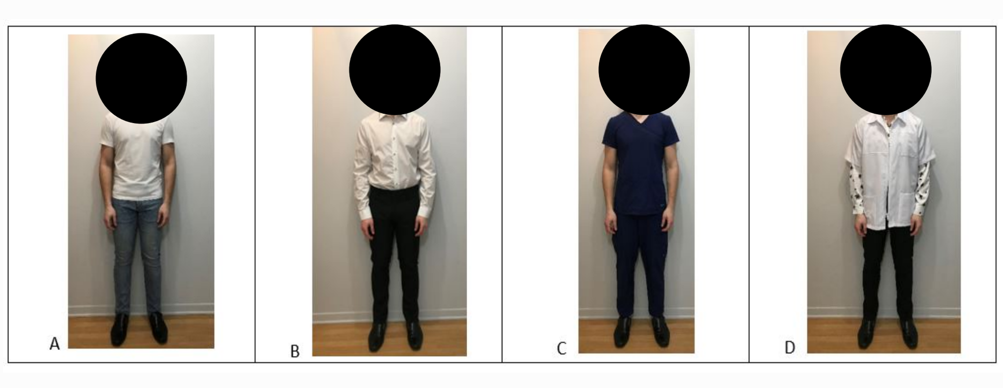

Classement

1.

- A
- B
- C
- D

Tenue préférée

2.

- A
- B
- C
- D

3.

- A
- B
- C
- D

4.

- A
- B
- C
- D

Tenue la moins préférée

SECTION 3 / 4 : SECTION C – GÉNÉRALITÉ SUR LA TENUE VESTIMENTAIRE

3.1. 1. La tenue vestimentaire du chiropraticien est importante pour moi

- Fortement en désaccord
- En désaccord
- Ni en accord ou désaccord
- D'accord
- Fortement en accord

2. La tenue vestimentaire du chiropraticien influence le niveau de satisfaction que j'ai pour mes soins

- Fortement en désaccord
- En désaccord
- Ni en accord ou désaccord
- D'accord
- Fortement en accord

3. Les chiropraticiens devraient porter un sarrau blanc quand ils voient des patients à la clinique

- Fortement en désaccord
- En désaccord
- Ni en accord ou désaccord
- D'accord
- Fortement en accord

SECTION 4 / 4 : SECTION D – DÉMOGRAPHIE

4.1. Quel âge avez-vous?

____

4.2. Quel est votre genre?

- Homme
- Femme
- Autres
- Je préfère ne pas répondre

4.3. Quel est le plus haut niveau de scolarité complété?

- Primaire
- Secondaire
- Cégep/Collège
- Université

4.4. Quelle description décrit le mieux votre ethnicité ou votre race?

- Blanc - Nord-Américain (Canadien, Américain)
- Blanc – Européen (Anglais, Italien, Portugais, Russe)
- Moyen - Orient (Égyptien, Iranien, Libanais)
- Latino (Argentin, Chilien, Salvadorien)
- Asiatique – Est (Chinois, Japonais, Coréen)
- Asiatique – Sud (Indien, Pakistanais, Sri Lankais)
- Asiatique – Sud-Est (Malaisien, Philippien, Vietnamien)
- Noir – Nord-Américain (Canadien, Américain)
- Noir – Africain (Kényan, Somalien, Ghénien)
- Noir – Caraïbe (Barbadien, Jamaïcain)
- Premières Nations
- Inuit
- Métis
- Ne préfère pas répondre
- Autre
